# Phytochemical Analysis, Antispasmodic, Myorelaxant, and Antioxidant Effect of *Dysphania ambrosioides* (L.) Mosyakin and Clemants Flower Hydroethanolic Extracts and Its Chloroform and Ethyl Acetate Fractions

**DOI:** 10.3390/molecules26237300

**Published:** 2021-12-01

**Authors:** Fahd Kandsi, Raffaele Conte, Mohamed Marghich, Fatima Zahra Lafdil, Mohamed F. Alajmi, Mohamed Bouhrim, Hamza Mechchate, Christophe Hano, Mohammed Aziz, Nadia Gseyra

**Affiliations:** 1 Laboratory of Bioresources, Biotechnology, Ethnopharmacology and Health, Faculty of Sciences, Mohammed First University, B.P. 717, Oujda 60000, Morocco; Kandsifahd1994@gmail.com (F.K.); Marghich.mohamed@ump.ac.ma (M.M.); Lafdil.fatimazahra@ump.ac.ma (F.Z.L.); mohamed.bouhrim@gmail.com (M.B.); Aziz.mohammed@ump.ac.ma (M.A.); 2 Research Institute on Terrestrial Ecosystems (IRET)—CNR, Via Pietro Castellino 111, 80131 Naples, Italy; Raffaele.conte86@tiscali.it; 3 Department of Pharmacognosy, College of Pharmacy, King Saud University, Riyadh 11451, Saudi Arabia; malajmii@ksu.edu.sa; 4 Laboratory of Biotechnology, Environment, Agrifood, and Health, Faculty of Sciences Dhar El Mahraz, University Sidi Mohmed Ben Abdellah, B.P. 1796, Fez 30000, Morocco; 5 Laboratoire de Biologie des Ligneux et des Grandes Cultures, INRAE USC1328, University of Orleans, CEDEX 2, 45067 Orléans, France; Christophe.hano@univorleans.fr

**Keywords:** antispasmodic, rat jejunum, rabbit jejunum, carbachol, verapamil, extraction, fractionation, LC/MS-MS, *Chenopodium ambrosioides*

## Abstract

*Dysphania ambrosioides* (L.) Mosyakin and Clemants is an annual or ephemeral perennial herb used traditionally in the Mediterranean region in folk medicine to treat various illnesses, including those related to the digestive system. This study aims to assess the antispasmodic, myorelaxant, and antioxidant effects of *D. ambrosioides* flower hydroethanolic extract and its chloroform and ethyl acetate fractions in a comparative study to evaluate the result of the extraction type on the potential activity of the extract. Both rat and rabbit jejunum were used to evaluate the antispasmodic and myorelaxant effect, while the antioxidant effect was evaluated using DPPH, a ferric reducing power assay, and a beta-carotene bleaching test. LC/MS-MS analysis was carried out to reveal the composition of the different types of extract. Following the results, the hydroethanolic extract showed a significant myorelaxant effect (IC_50_ = 0.39 ± 0.01 mg/mL). Moreover, it was shown that the hydroethanolic extract demonstrated the best antispasmodic activity (IC_50_ = 0.51 ± 0.05 mg/mL), followed by the ethyl acetate (IC_50_ = 4.05 ± 0.32 mg/mL) and chloroform (IC_50_ = 4.34 ± 0.45 mg/mL) fractions. The antioxidant tests showed that the hydroethanolic extract demonstrated high antioxidant activity, followed by the ethyl acetate and chloroform fractions. The LC/MS-MS analysis indicates that the plant extract was rich in flavonoids, to which the extract activity has been attributed. This study supports the traditional use of this plant to treat digestive problems, especially those with spasms.

## 1. Introduction

*Dysphania ambrosioides* (L.) Mosyakin and Clemants*—*formerly *Chenopodium ambrosioides*, otherwise known as Jesuit’s tea, Mexican tea, payqu (paico), epazote, mastruz, or herba sanctæ Mariæ (Arabic: M’khinza, French: anserine vermifuge)—is a wild species from tropical America naturalized in the Old World [1]. The World Health Organization (WHO) reported that *D. ambrosioides* is among the most widely used medicinal plants [2]. It can be used as a antirheumatic, analgesic [3], sedative, and antipyretic [4]. It is used as an herbal tea in Mexico to stimulate milk production and to improve blood flow. This plant has been widely used as a remedy for digestive disorders since centuries ago [5]. In Morocco, the entire plant is used as an infusion or juice for gastrointestinal diseases, typhoid, and dysentery in children and adults and as a galactogen. It is also used against oral abscesses, ulcerations, and purulent wounds by local application of the fresh plant [6]. Several authors have described the antioxidant [7], anti-leishmaniasis [8], antitumor [9], anthelmintic [10], molluscicidal [11], nematocidal [12], antimalarial [13], anti-inflammatory [14], and insecticidal properties of this plant [15,16,17].

The diverse parts of the plant are known to contain many phytochemical compounds. Thirty-five compounds have been discovered in *D. ambrosioides,* especially phenolic compounds [18]. Among the most abundant phenolic compounds are flavonoids (quercetin and kaempferol), terpenoids, and phenolic acids. They are known for their potent antioxidant properties and beneficial effect on gastrointestinal problems [19,20]. Gastrointestinal problems are among the most recurrent and confronted illnesses in humans. Functional bowel disorders are chronic digestive symptoms that indicate the digestive tract’s dysfunction, especially the small intestine and the colon, and no organic abnormality can explain it [21].

The aerial parts of *D. ambrosioides*, including the flowers, are traditionally used in Tunisia as a digestive and carminative [22]. In Brazil, the essential oil extracted from the fresh inflorescence of this plant is used against the necrotrophic fungus *Botrytis cinerea* [23]. In the Dominican Republic, the flowers have been reported to be used to regulate menses [24]. In Morocco, the plants’ aerial parts have been reported to treat acute digestive system aches in the Region of Fez-Meknes [25].

Taking into account that local traditional health practitioners in the North-Eastern region of Morocco recommend using the plant at its flowering stage, especially the flowers to treat digestive related diseases, this study was undertaken to explore and validate the traditional use of this plant, specifically those of the flowers. The antispasmodic and myorelaxant effects of *D. ambrosioides* flower hydroethanolic extract and its chloroform and ethyl acetate fractions were evaluated using rat and rabbit Jejunum (ex vivo). The antioxidant potential was assessed using 2,2-diphényl-1-picrylhydrazyl (DPPH), a ferric reducing power assay (FRAP), and a beta carotene bleaching test. The composition of the hydroethanolic extract and the fractions were analyzed using an LC/MS-MS method.

## 2. Results

### 2.1. Yield of Extractions

**Figure 1:**
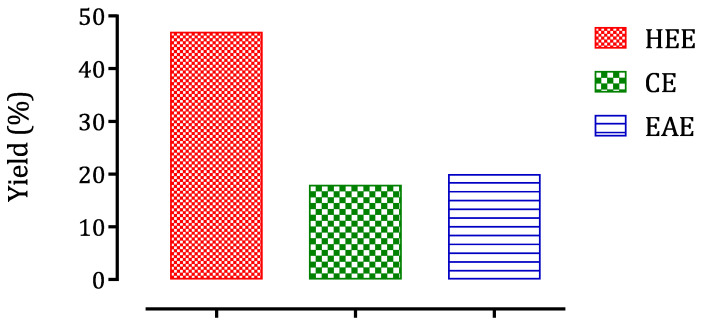
The yield of the hydroethanolic extract and the chloroform and ethyl acetate fractions. HEE—hydroethanolic extract; CE—chloroform extract; EAE—ethyl acetate extract.

The extraction and fractionation yields are summarized in Figure 1. The yield of crude hydroethanolic extract was 47%, while fractions yielded 18% and 20% for chloroform and ethyl acetate, respectively. 

### 2.2. Phytochemical Analysis 

**Figure 2:**
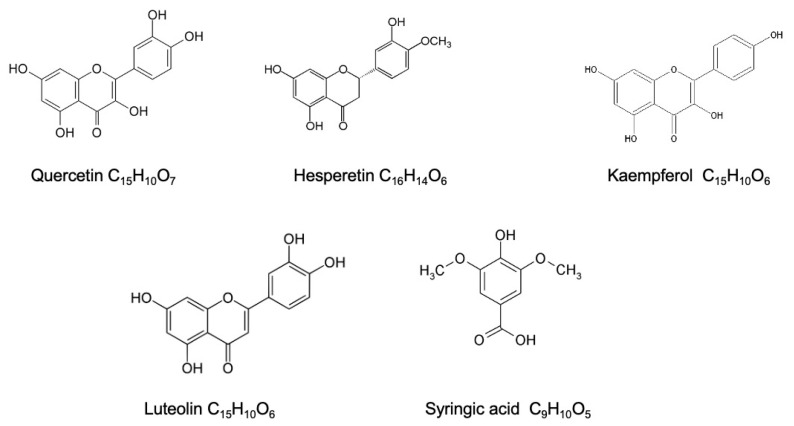
Most abundant molecules in the *D. ambrosioides* extract.

The chromatographic analysis of the different fractions revealed the existence of various compounds. The hydroethanolic extract contains mainly syringic acid, quercetin, hesperetin, and luteolin. The ethyl acetate fraction contains mainly kaempferol, syringic acid, quercetin, hesperetin, luteolin, and salicylic acid. The chloroform fraction contains kaempferol-3*-O-*pentoside, rosmarinic acid, trimethoxyflavone, syringic acid, and quercetin. The three extracts contain some similar compounds (Figure 2); this is because the ethyl acetate and chloroform are portions of the hydroethanolic extract. The chemical characteristics of the three solvents are different, and the ability of one solvent to attract a compound is different from others. Most molecule families presented in the extracts were flavonoids and phenolic acids, with the ethyl acetate fraction being the richest in flavonoids, as shown in the screening results below (Table 1).

### 2.3. Antioxidant Activity of D. ambrosioides Extract and Fractions

The IC_50_ values of DPPH free radical scavenging for the hydroethanolic extract and chloroform and ethyl acetate fractions are 166.47 ± 1.74 µg/mL, 1043 ± 1.04 µg/mL, and 156.8 ± 0.96 µg/mL, respectively (Table 2). These results indicate that the hydroethanolic extract’s DPPH free radical scavenging activity is closer to that of the ethyl acetate fraction and ascorbic acid. However, the chloroform fraction has a lower DPPH free radical scavenging activity than the other samples. The results of the β-carotene bleaching test showed that the IC_50_ of the hydroethanolic extract and chloroform and ethyl acetate fractions are 57.04 ± 0.06 µg/mL, 66.01 ± 1.00 µg/mL, and 60.1 ± 1.06 µg/mL respectively. These results show that the plant extract and fractions have approximatively the same inhibitive effect against β-carotene bleaching. Moreover, this effect is lower than the BHT. The results of the ferric reducing power test show that the hydroethanolic extract has greater ferric reducing power, with an IC_50_ of 231.5 ± 0.25 µg/mL, followed by the ethyl acetate fraction, with an IC_50_ of 511.8 ± 0.30 µg/mL, while the chloroform fraction has the weakest ferric reducing power, with a high IC_50_ of 1017.8 ± 0.57 µg/mL. Ascorbic acid has great ferric reducing power compared to the plant extract and fractions.

### 2.4. Antispasmodic and Myorelaxant Activity of D. ambrosioides Extract and Fractions

#### 2.4.1. Antispasmodic Effect of the Extract and Fractions on the Tone of the Rat Jejunum Induced by CCh (Carbachol)

**Figure 3:**
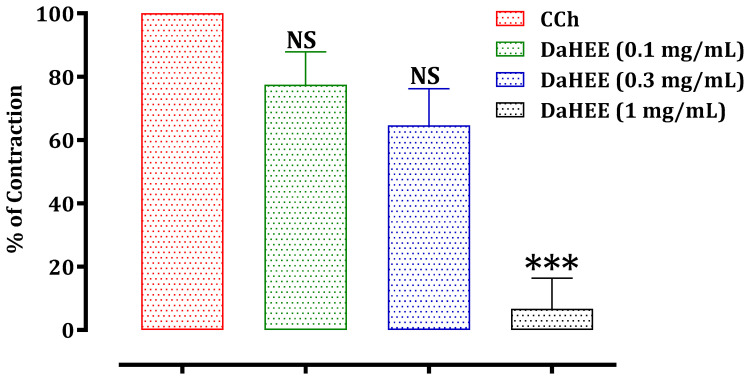
Effect of the DaHEE on rat jejunum precontracted by carbachol 10^−6^ M. NS: not significant; *** *p* ≤ 0.001 in comparison with the control. The values are the means ± SEM (*n* = 5). DaHEE—*D.* *ambrosioides* hydroethanolic extract; CCh—carbachol.

**Figure 4:**
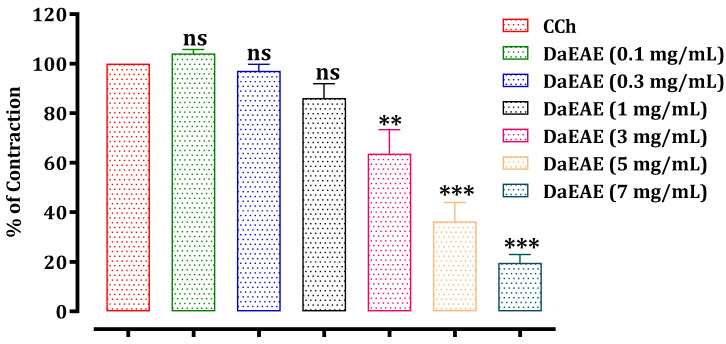
Effect of the DaEAE on rat jejunum precontracted by carbachol 10^−6^ M. ns: not significant; ** *p* ≤ 0.01 and *** *p* ≤ 0.001 in comparison with the control. The values are the means ± SEM (*n* = 5). DaEAE—*D.* *ambrosioides* ethyl acetate extract; CCh—carbachol.

**Figure 5:**
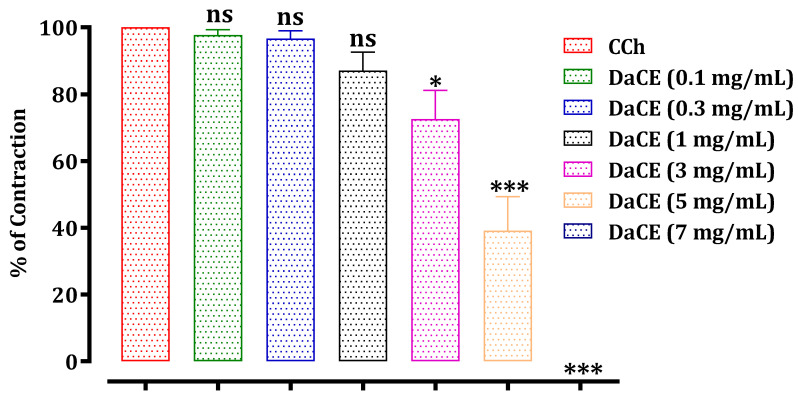
Effect of the DaCE on rat jejunum precontracted by carbachol 10^−6^ M. ns: not significant; * *p* ≤ 0.05 and *** *p* ≤ 0.001 compared to the control. The values are the means ± SEM (*n* = 4). DaCE—*D.* *ambrosioides* chloroform extract; CCh—carbachol.

The results of this test are presented in Figure 3, Figure 4 and Figure 5. The hydroethanolic extract of *D. ambrosioides* caused a significant inhibition of the rat jejunum contraction induced by carbachol 10^−6^ M in a dose-dependent way, with an IC_50_ of 0.51 ± 0.05 mg/mL. The ethyl acetate and chloroform fractions of *D. ambrosioides* significantly inhibited the contraction induced by CCh in a dose-dependent way, with IC_50_ values of 4.05 ± 0.32 mg/mL and 4.34 ± 0.45 mg/mL, respectively. The hydroethanolic extract exhibited a greater antispasmodic effect than the ethyl acetate and chloroform fractions.

#### 2.4.2. Myorelaxant Effect of *D. ambrosioides* Hydroethanolic Extract on the Spontaneous Contraction of Rabbit Jejunum

**Figure 6:**
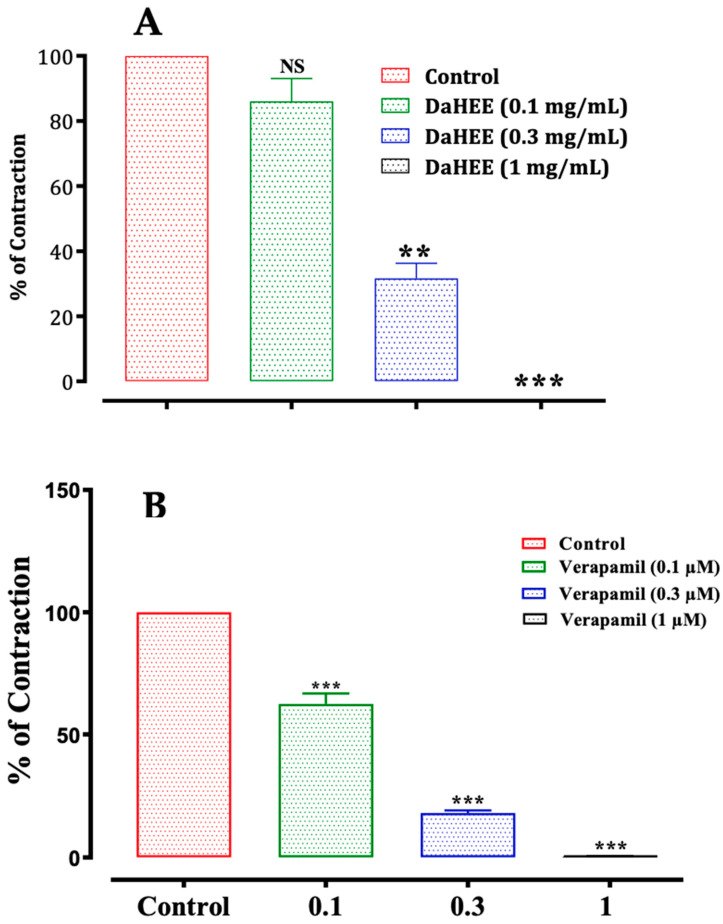
Myorelaxant effect of DaHEE flowers(**A**) and verapamil (**B**) and the on spontaneous contractions of the rabbit jejunum. NS: not significant; ** *p* ≤ 0.01 and *** *p* ≤ 0.001 compared to the control. The values are the means ± SEM (*n* = 6). DaHEE—*D. ambrosoidese* hydroethanolic extract.

This test demonstrated that the hydroethanolic extract of *D. ambrosioides* has a myorelaxant effect through the inhibition of the basal contractions of rabbit jejunum in a dose-dependent way, with an IC_50_ of 0.39 ± 0.01 mg/mL. Moreover, the hydroethanolic extract induced a significant myorelaxant effect at the doses of 0.3 (*p* ≤ 0.01) and 1 (*p* ≤ 0.001) mg/mL, in comparison with the control test (spontaneous contraction) (Figure 6A). Verapamil (1 µM), which is an antagonist of L-type calcium channel blockers, had an effect comparable to that of 1 mg/mL of hydroethanolic extract (Figure 6B).

## 3. Discussion

In this comparative study, we tried to reveal the activity of different types of extract prepared from the flowers of *D. ambrosioides* in relation to antioxidant, antispasmodic, and myorelaxant properties. The solvent choice affected the composition of the extract. In the antioxidant test, the hydroethanolic extract exhibited the best activity during the DPPH, FRAP, and β-carotene tests. Similarly, the hydroethanolic extract exhibited the best activitiy during the antispasmodic and myorelaxant tests performed on rat and rabbit jejunum. The hydroethanolic extract was prepared using a solid–liquid extraction (SLE), which is the best way to produce extracts with the goal of industrialization due to the low technical and facility requirements, despite the development of more recent and efficient ways (novel extraction procedures) marked by more advanced technologies that lead to increased productivity and lower environmental impact [26,27]. Organic solvents—for instance, ethanol and acetone—are the most often utilized due to their safety and cheap cost. The cost of evaporation and the capacity to extract specific compounds are all factors that affect the solvent choice [28]. For all of these reasons, binary mixtures of water and ethanol are often used to produce natural extracts for industry [29,30]. The advantages of hydroethanolic preparations, as well as their safety and effectiveness, make it the best option for any future preparation to treat illnesses linked to intestinal problems, particularly spasms.

In this study, the hydroethanolic extract of the flowers of *D. ambrosioides* and its chloroform and ethyl acetate fractions demonstrated significant antioxidant activity. These results are consistent with those presented by Barros et al. [28]; they found that the infused and methanolic extract of this plant demonstrated good antioxidant activity. To the best of our knowledge, no reports are available on the antispasmodic effect of the flowers of *D. ambrosioides*.

The phytochemical analysis revealed that the majority of the components of the three extracts were from flavonoids, phenolic acids, and other polyphenols. In several antioxidant studies, polyphenols have been found to have a high degree of free radical scavenging activity [31,32]. Polyphenols are a diverse category of compounds, with one or more hydroxyl groups. Polyphenols may take many different forms, ranging from simple phenolic acids to more complex tannins. The ability of a polyphenol, like all other antioxidants, to scavenge free radicals is determined by its structure [31]. The most significant variables are the number of OH groups and the degree of methoxylation. Polyphenolic activity is also linked to the capacity of the phenolic structure to promote metal ion chelation to scavenge alkoxyl and peroxyl radicals [33,34]. The strong free radical scavenging activity of phenolic acids is determined by the quantity and relative location of the OH groups attached to the aromatic ring. Flavonoids have been shown to chelate metals via a variety of mechanisms, including the formation of reducing radicals such as superoxide, peroxyl, alkoxyl, and hydroxyl through hydrogen donation [35].

Carbachol (CCh) is an agonist of acetylcholine (Ach) that is not degraded by acetylcholinesterase, provoking the contraction of the intestine by acting on M2 and M3 muscarinic receptors [18]. M2 acts via the inhibition of adenylate cyclase, whilst M3 acts via phospholipase C and Gq/11 G proteins to increase Ca^2+^ entry and release from the sarcoplasmic reticulum. Muscarinic antagonists impede the contractions of the gastrointestinal tract induced by Ach and other muscarinic agonists such as CCh [20]. This was the case in our study, demonstrated by the fact that the different extracts of our plant stopped the contraction induced by CCh in a dose-dependent way, but with different efficiencies. We can suggest that the *D.* *ambrosioides* may contain components that have a cholinergic receptor blocking effect and act on M2 and M3 muscarinic receptors.

The origin of spontaneous phasic contraction is electrical slow waves, which are cycles of depolarization/repolarization, which result from the intrinsic activity of a pacemaker. The activity caused by this phenomenon is electrically generated and spread to muscle cells through Cajal interstitial cells (ICC) [19]. The basic spontaneous contractions of the rabbit jejunum are larger and easier to assess than those of the rat jejunum. The hydroethanolic extract of *D.* *ambrosioides* caused a decrease in the amplitude of spontaneous contractions of the smooth muscle of the jejunum. The dose of 1 mg/mL demonstrated a total inhibition of this contraction. This effect is comparable with verapamil, which is an antagonist of L-type voltage-dependent calcium channel blockers. Therefore, the hydroethanolic extract of *D. ambrosioides* has a myorelaxant effect on rabbit jejunum base contractions in a dose-dependent way, by blocking calcium channels or by their effect on the origin of this spontaneous contraction, which is the electrical slow waves and ICC.

The spasmolytic activity of different extracts of our plant can be explained by the existence of different flavonoids such as quercetin and luteolin, which have previously been characterized by high antispasmodic activity, with IC_50_ values of 7.8 mol/L and 9.8 μmol/L, respectively [36]. Quercetin also reduced the contraction induced by CCh in isolated mouse stomachs in a concentration-dependent way. In addition, this molecule induced a concentration-dependent relaxation of the stomach and concentration-dependent inhibition of the contractile responses resulting from the exogenous application of Ca^2+^ in a Ca^2+^-free solution with a high concentration of K^+^, implying that the gastric myorelaxant effects of quercetin are due to their negative modulation of calcium influx through voltage-dependent Ca^2+^ channels [37]. Hesperetin is known for its antispasmodic effect mediated by fast-current low-voltage activated K^+^ channels as well as voltage-independent K^+^ channels via the nitric oxide pathway [38]. Our result indicates that the hydroethanolic extract has a better effect in comparison with the two other fractions, perhaps due to the presence of caffeic acid and rutin, which caused the inhibition of spontaneous contraction and also inhibited the low- and high-potassium medium-induced contractions [39]. In terms of plant activity, Pereira-de-Morais et al. found that *D. ambrosioides* essential oil and its main component, α-terpinene, had a myorelaxant effect on rat tracheal smooth muscle, which may be caused by the inhibition of the L-type calcium channel, which blocks the inward Ca^2+^ current via these channels, although this does not rule out the potential of additional processes being involved [40]. 

## 4. Materials and Methods

### 4.1. Chemicals and Reagents

The following substances were used in this study: carbamylcholine chloride (carbachol, CCh), Folin–Ciocalteau, ascorbic acid, gallic acid, β-carotene, rutin, DPPH (1,1-diphenyl-2-picrylhydrazyl), sodium hydroxide (NaOH), sodium nitrate (NaNO_3_), potassium ferricyanide (K_3_Fe(CN)_6_), trichloroacetic acid (TCA), aluminum chloride (AlCl_3_), methanol, ethanol, chloroform, acetate ethyl, sodium phosphate (Na_3_PO_4_), monobasic sodium phosphate (NaH_2_PO_4_), ferric chloride (FeCl_3_), dibasic sodium phosphate (Na_2_HPO_4_). These substances were all acquired from Sigma-Aldrich Chemicals (St. Louis, MO, USA), and each chemical was of analytical grade.

### 4.2. Harvest and Preparation of the Plant

The *D. ambrosioides* flowers were collected in Guercif (North-Eastern Morocco). Professor Abdelbasset Berrichi of the Biology Department of the Faculty of Science (Oujda, Morocco) carried out the botanical identification of this plant, where a specimen of the voucher was set under collection number ZL28.

### 4.3. Extraction and Fractionation

After drying the plant at room temperature, 450 g of the flowers (ground) were subjected to maceration by ethanol (70%) at room temperature and put under agitation for a week. After removing the solvent, a portion of the hydroethanolic extract was dried to a temperature of 30 °C, resulting in the dry crude extract. The remaining hydroethanolic extract was fractionated using two increasingly polarized solvents (chloroform and ethyl acetate) to produce two fractions, chloroform and ethyl acetate.

The yield of the extraction was determined by calculating the ratio between the weight of the dry extract and the weight of the plant material used for extraction in grams (Equation (1)).

(1) $$\mathrm{Yield}\;\left(\%\right)=\frac{\mathrm{Wextract}\;}{\mathrm{Wsample}}\times 100$$

W_extract_: extract weight in grams. 

W_sample_: sample weight (plant) in grams.

### 4.4. LC-MS/MS Analysis of the Extract and Its Fraction Components 

Aliquots of samples (80 mg) were treated according to the following extraction procedure: The aliquot was treated with 1 mL of ethanol. The Eppendorf was vortexed and incubated in a sonicator bath at 45 °C for 60 min. Qualitative analysis was carried out using a Shimadzu Ultra-High-Performance Liquid Chromatograph (Nexera XR LC 40, Kyoto, Japan), which was combined with an MS/MS detector (LCMS 8060, Shimadzu Italy, Milan, Italy). The MS/MS was employed with electrospray ionization and controlled by Lab Solution software (ver. 5.6, Kyoto, Japan), which facilitated fast change from a low-energy scan 4 V (full scan MS) to a high-energy scan (10–60 V ramping) during a single LC run.

The source parameters were fixed as follows: nebulizing gas flow, 2.9 L/min; heating gas flow, 10 L/min; interface temperature, 300 °C; DL temperature, 250 °C; heat-block temperature, 400 °C; and drying gas flow, 10 L/min. Flow injection was used to perform the analysis (meaning that there was no chromatographic separation), with a mobile phase consisting of acetonitrile and water + 0.01% formic acid in a ratio of 5:95 (*v/v*). The instrument was set for a SIM experiment in negative mode (only syringic acid for positive ESI) [41,42]. The identification of the molecules was confirmed by comparing the typical fragment identified with those in our in-house-developed library of molecules, and a molecule was considered positive if its area under the curve was higher in magnitude than those of the blank (the Supplementary Materials Files S1–S4 contain the data of the molecules’ retention time and typical fragment m/z). Differentiation between very similar structures was done via time of flight, as the instrument was set to acquire the molecular weight in the third quadrupole.

### 4.5. Antioxidant Potential of D. ambrosioides Hydroethanolic Extract and Its Chloroform and Ethyl Acetate Fractions

#### 4.5.1. Scavenging 1,2-Diphenyl-1-Picrylhydrazyl (DPPH) Radical Capacity

The capacity of our plant to trap the DPPH radical was tested by the method described by Bouhrim et al., with some modifications [43]. After a concentration series (25, 50, 100, 200, 400, 800, 1000 µg/mL) of the tested extracts had been prepared, the reaction mixture comprised 2 mL of the plant extract and 1 mL of DPPH ethanolic solution (0.004%). The mixture was vortex shaken and left for 30 min for incubation. After that, a spectrophotometer immediately determined the absorbance at 517 nm. As for the positive control (Ascorbic acid (AA)), the same experimental procedure was used), a standard antioxidant. All of the tests were performed in triplicate. The results have been expressed in terms of the scavenging percentage and the inhibitory concentration (IC_50_), which is the effective concentration of the extract that can trap 50% of the radicals (DPPH) in the reaction mixture. Sample scavenging activity was calculated as follows (Equation (2)):

DPPH scavenging percentage (%) = (AB_DPPH_ − AB_SAMPLE_/AB_DPPH_) × 100 (2)

where AB_DPPH_ corresponds to the absorbance of DPPH solution without the sample, and AB_SAMPLE_ corresponds to the absorbance of the test sample mixed with the DPPH solution. 

#### 4.5.2. β-Carotene Bleaching Test

To examine the antioxidant activity, this test was performed using the method described by Miller in 1971 [44]. Briefly, an emulsion of β carotene/linoleic acid was prepared by solubilizing 2 mg of β-carotene in 1000 µL of chloroform, and then 2 mg of linoleic acid and 200 mg of Tween 40 were added. Chloroform was then evaporated using a rotavapor, and 100 mL of oxygenated distilled water was added, with vigorous stirring of the resulting emulsion. For this test, 2500 µL of the previous emulsion was added to a series of tubes containing 5 µL of the extract to be analyzed. BHA was used as a positive control. Absorbance values were read before and after 2 h of incubation at 492 nm. The measurements were made in triplicate. The following formula (Equation (3)) was used for the calculation of the inhibition of the lineolate radical/β-carotene:

(3) $$\;\mathrm{Bleaching}\;\mathrm{inhibition}\;\%=\frac{\mathrm{initial}\;\beta \;\mathrm{carotene}-\;\beta \;\mathrm{carotene}\;\mathrm{after}\;2h}{\mathrm{initial}\;\beta \;\mathrm{carotene}}\times 100$$

The IC_50_, which is the effective concentration of the extract that can inhibit 50% bleaching of β-carotene, was calculated.

#### 4.5.3. The Ferric Reducing Power Assay (FRAP)

The iron reducing activity of the extracts was calculated using the method described by Jelena Katanić et al. [45]. Distinct concentrations of the extracts were prepared. Volumes of 0.5 mL of each specimen extract were added to 1.25 mL phosphate buffer (0.2 M, pH 6.6) and 1.25 mL potassium ferricyanide [K_3_Fe (CN) 6] (1% *w/v*). Subsequently, the combination was incubated for 20 minutes at 50 °C. After cooling to room temperature, the reaction was ceased by the addition of 1.25 mL of trichloroacetic acid (10% *w/v).* Later, the mixture was centrifuged at 3000 rpm for exactly 10 min. An aliquot of 1.25 mL of the supernatant solution was combined with 1.25 mL of distilled water and 0.25 mL of ferric chloride solution (0.1% *w/v*), and the absorbance at 700 nm was measured by spectrophotometry. Ascorbic acid was used as a reference compound. All of the tests were performed in triplicate. The concentration of the 0.5 absorbance sample (IC_50_) was calculated by setting the absorbance at 700 nm with respect to the corresponding concentration of the sample.

### 4.6. Antispasmodic Activity of D. ambrosioides Hydroethanolic Extract and Its Chloroform and Ethyl Acetate Fractions

#### 4.6.1. Animals

These experiments were carried out on both female and male New Zealand rabbits (1.5–2 kg) and Wistar rats (200–300 g). The animals were retained in a room with an air-conditioner, controlled lighting (12 h/12 h light/darkness cycle), with free access to food and water, in the animal house of the faculty of sciences, Oujda, Morocco. Food was taken out 24 h before the experiment. All animals were taken care of in compliance with the internationally accepted guide for the care and use of laboratory animals published by the US National Institutes of Health [46].

#### 4.6.2. Isolated Tissue Experiments

After having anesthetized the animal, the abdominal cavity was opened, and 2 cm jejunum fragments were removed and preserved throughout the tests in the oxygenated normal Krebs–Henseleit buffer (KHB) solution (in mM): NaCl, 118; KCl, 4.7; CaCl_2_, 2.5; MgSO_4_, 1.2; NaHCO_3_, 25; KH_2_PO_4_, 1.2; and glucose, 10. The KHB solution was maintained at a temperature of 37 ° C and a pH of 7.4 with continual oxygenation for 1 h to have the same physiological conditions as the animal. Each piece of jejunum was mounted in an isolated organ tank. Every 15 min, the physiological fluid was changed to balance the organ before adding the plant extracts or other drugs. The effects of each dose were recorded for at least 7 to 8 min for all experiments.

#### 4.6.3. Myorelaxant Activity on Isolated Rabbit Jejunum

After stabilization of baseline contractions of rabbit jejunum, cumulative doses (0.1–1 mg/mL) of the hydroethanolic extract of *D*. *ambrosioides* flowers were added to the isolated organ chamber.

#### 4.6.4. Antispasmodic Test

We pre-contracted jejunal smooth muscle through the application of CCh 10^−6^ M. After stabilization, cumulative doses of the different extracts were added to the isolated organ tank. The IC_50_ has been calculated to better compare the effect of the different extracts.

### 4.7. Statistical Analysis

The results have been expressed as the mean ± S.E.M. Moreover, the difference between the groups has been calculated with a one-way analysis of variance (ANOVA) using GraphPad Prism (Ver. 5, San Diego, CA, USA) for Windows, followed by a post-hoc Tukey test. We consider the difference to be significant when *p* is less than 5%.

## 5. Conclusions

This comparative study proved that the prepared hydroethanolic extract from *D. ambrosioides* flowers exhibits the best antioxidant, antispasmodic, and myorelaxant effect compared to those obtained from other fractions, such as ethyl acetate and chloroform. Our results also show that the tested extracts of *D. ambrosioides* flowers are rich in total polyphenols and flavonoids, and that these would be at least partially responsible for all their activities. It is not excluded that other classes of compounds could also are be involved, such as tannins and alkaloids among others. The hydroethanolic extract is the closest preparation to that made in traditional medicine. This solvent has the property of extracting the most active molecules contained in vegetable matrices. This study also encourages and supports the traditional usage of the flowers of *D. ambrosioides* to treat the studied illnesses (oxidative stress and gastrointestinal disorders).

## Figures and Tables

**Table 1:** Phytochemical composition of the extract and fractions revealed by LC-MS/MS.

Molecule	Hydroethanolic Extract	Ethyl Acetate Fraction	Chloroform Fraction
Catechin gallate (epicatechin gallate)	−	+	+
Amentoflavone	−	+	+
Arbutin	−	+	−
Caffeic acid	+	−	−
Ferulic acid	−	+	−
Gallocatechin/Epigallocatechin gallate	−	+	+
Hesperetin	++	+++	+
Isorhamnetin-3-*O*-rutinoside	−	+	+
Isorhamnetin-7-*O*-pentoside	+	+	+
Kaempferol	++	+++	−
Kaempferol-3-*O*-glucoside	+	−	+
Kaempferol-3-*O*-glucuronic acid	−	+	+
Kaempferol-3-*O*-hexose deoxyhexoside	+	+	+
Kaempferol-3-*O*-pentoside	+	+	+++
Luteolin	++	++	−
Luteolin 7-*O*-glucoside	+	+	+
Myricetin	−	+	+
Naringin	+	−	−
Oleochantal	−	+	−
*p*-coumaric acid	+	−	−
*p*-hydroxybenzoic/Salicylic acid	+	++	−
Procyanidin	+	+	−
Quercetin	++	+++	++
Quercetin-3-*O*-glucoside	−	+	+
Quercetin-3-O-glucuronoside	−	+	+
Quercetin-3-*O*-hexose deoxyhexoside	+	−	+
Rosmarinic acid	+	+	++
Rutin	+	−	−
Syringic acid	+++	+++	+++
*Trans* ferulic acid	+	+	−
Trimethoxyflavone	−	+	++
Ursolic acid	−	+	+

+++: High abundance, ++: abundant, +: low abundance, −: not detectable in the extract.

**Table 2:** Antioxidant activity of *D. ambrosioides* extract and fractions.

Samples	IC_50_ (µg/mL)		
	DPPH	β-Carotene	FRAP
Hydroethanolic extract	166.47 ± 1.74 ^NS^	57.04 ± 0.06 ^≠^	231.5 ± 0.25 ^NS^
Chloroform fraction	1043 ± 1.04 ***	66.01 ± 1.00 ^≠≠^	1017.8 ± 0.57 **
Ethyl acetate fraction	156.8 ± 0.96 ^NS^	60.1 ± 1.06 ^≠^	511.8 ± 0.30 ^NS^
Ascorbic acid	137.7 ± 0.05	-	33.7 ± 0.01
BHA		32.02 ± 2.6	

BHA—butylated hydroxyanisole; DPPH—2,2-diphényl-1-picrylhydrazyl; FRAP—ferric reducing power assay. NS: not significant; ** *p* ≤ 0.01 and *** *p* ≤ 0.001 in comparison with the ascorbic acid; **^≠^** *p* ≤ 0.05 and **^≠≠^** *p* ≤ 0.01 in comparison with the BHA.

## Data Availability

Data are available upon request.

## Supplementary Materials

The following are available online, File S1: LC/MS polyphenols analysis blank, File S2: LC/MS polyphenols analysis Chloroform extract, File S3: LC/MS polyphenols analysis Ethyl acetate extract, File S4: LC/MS polyphenols analysis Hydroethanolic extract.

## Sample Availability

Samples of the plants extract are available from the authors.
