# Supplementary material for: Phytochemical Analysis, Antispasmodic, Myorelaxant, and Antioxidant Effect of Dysphania ambrosioides (L.) Mosyakin and Clemants Flower Hydroethanolic Extracts and Its Chloroform and Ethyl Acetate Fractions
Source: Molecules. 2021 Dec 1;26(23):7300. doi: 10.3390/molecules26237300 (PMC8659140; doi:10.3390/molecules26237300)
Supplement: Supplementary file 1 [file molecules-26-07300-s001.zip › molecules-1444408-supplementary/File S1_blnk_polyphenols.pdf]

# = Shimadzu LabSolutions Quant. Browser Data Report =

Acquired by	: System Administrator
Data Acquired	: 23/06/2021 11:03:10
Sample Type	: Unknown
Sample Name	: blnk
Sample ID	:
Sample Amount	: 1
Dilution Factor	: 1
Vial#	: 1
Injection Volume	: 1 uL
Data Filename	: blnk_002.lcd
Method Filename	: polifenoli screening SIM.lcm
Processed by	: System Administrator
Modified Date	: 23/06/2021 11:56:36

198.9000(+)@1ID#:1 m/z: 198.9000

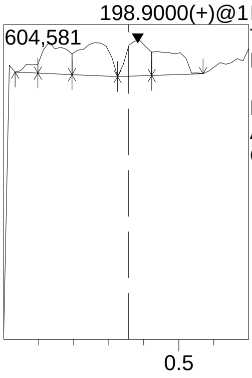

Type:Target
Name:syringic acid SIM

Ret.Time:0.383
Area:293680
Conc.:0.000ppm

168.9000(-)@2ID#:2 m/z: 168.9000

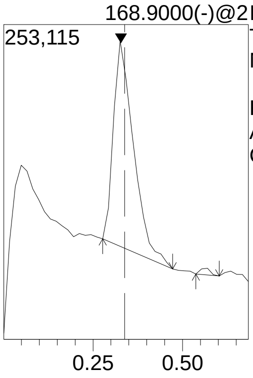

Type:Target
Name:gallic acid SIM

Ret.Time:0.328
Area:543889
Conc.:0.000ppm

301.0000(-)@3ID#:3 m/z: 301.0000

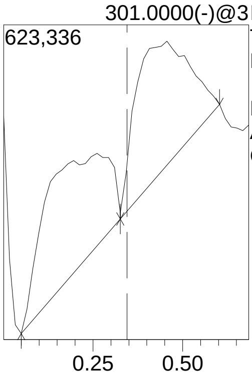

Type:Target
Name:quercetin sim

Ret.Time:0.000
Area:0
Conc.:N.D.(W/B)

162.9000(-)@4ID#:4 m/z: 162.9000

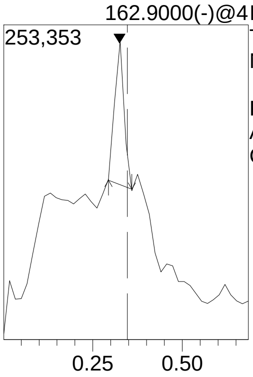

Type:Target
Name:p coumarci acid SIM

Ret.Time:0.325
Area:111418
Conc.:0.000ppm

303.2000(-)@5ID#:5 m/z: 303.2000

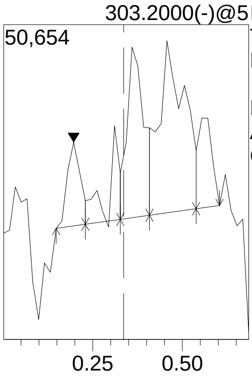

Type:Target
Name:oleochantal SIM

Ret.Time:0.197
Area:8181
Conc.:0.000ppm

153.0500(-)@6ID#:6 m/z: 153.0500

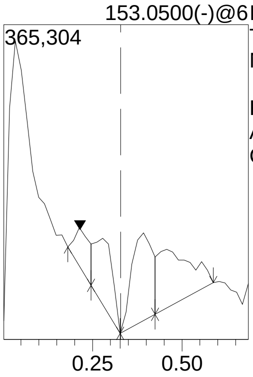

Type:Target
Name:hydroxytyrosol SIM

Ret.Time:0.215
Area:74490
Conc.:0.000ppm

193.0000(-)@7ID#:7 m/z: 193.0000

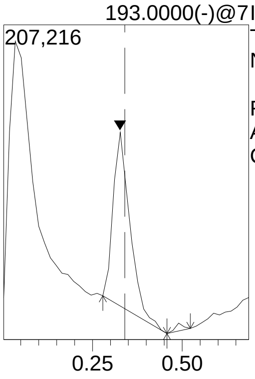

Type:Target
Name:trans ferulic acid SIM

Ret.Time:0.327
Area:260406
Conc.:0.000ppm

539.0000(-)@8ID#:8 m/z: 539.0000

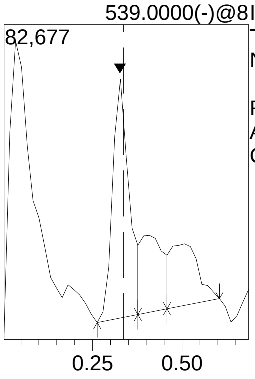

Type:Target
Name:oleuropein SIM

Ret.Time:0.327
Area:125695
Conc.:0.000ppm

301.3000(-)@9ID#:9 m/z: 301.3000

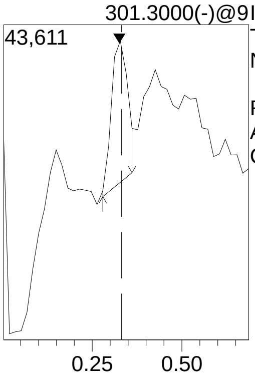

Type:Target
Name:Hesperetin

Ret.Time:0.325
Area:36038
Conc.:0.000ppm

312.0000(-)@10ID#:10 m/z: 312.0000

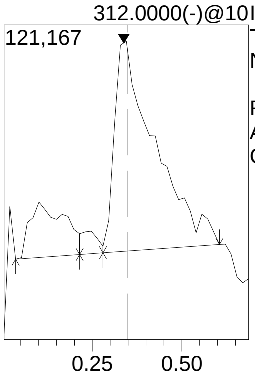

Type:Target
Name:trimethoxyflavone

Ret.Time:0.338
Area:311090
Conc.:0.000ppm

271.2000(-)@11ID#:11 m/z: 271.2000

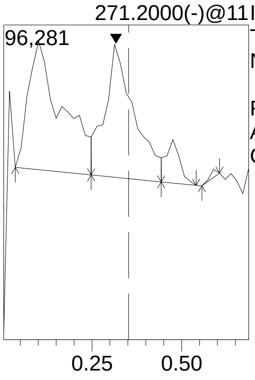

Type:Target
Name:Arbutin

Ret.Time:0.317
Area:125903
Conc.:0.000ppm

359.0000(-)@12ID#:12 m/z: 359.0000

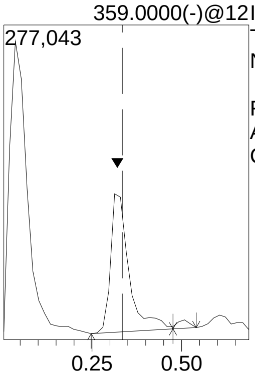

Type:Target
Name:Rosmarinic acid

Ret.Time:0.321
Area:305317
Conc.:0.000ppm

455.0000(-)@13ID#:13 m/z: 455.0000

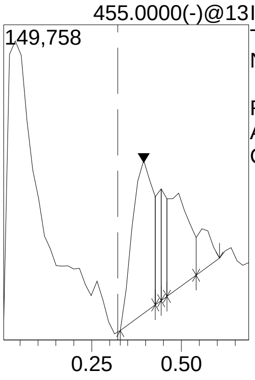

Type:Target
Name:ursolic acid

Ret.Time:0.395
Area:130518
Conc.:0.000ppm

269.0000(-)@14ID#:14 m/z: 269.0000

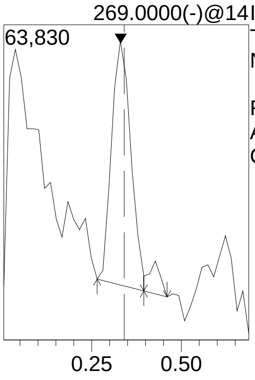

Type:Target
Name:Apigenin

Ret.Time:0.331
Area:67176
Conc.:0.000ppm

537.1000(-)@15ID#:15 m/z: 537.1000

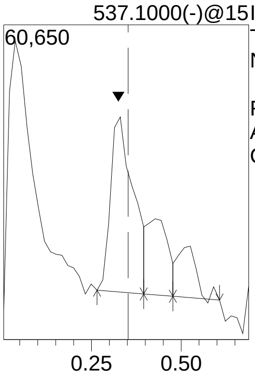

Type:Target
Name:Amentoflavone

Ret.Time:0.325
Area:82841
Conc.:0.000ppm

284.9000(-)@16ID#:16 m/z: 284.9000

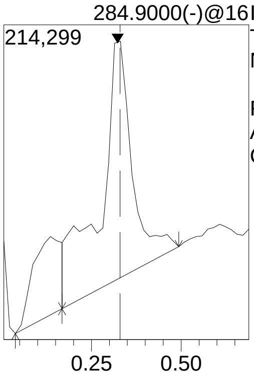

Type:Target
Name:luteoilin

Ret.Time:0.323
Area:677963
Conc.:0.000ppm

463.1000(-)@17ID#:17 m/z: 463.1000

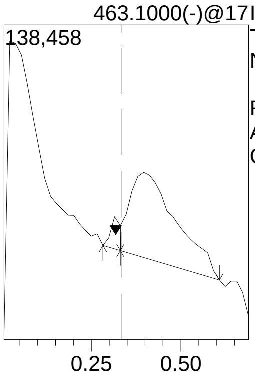

Type:Target
Name:quercetin-3-O-glucoside

Ret.Time:0.318
Area:15571
Conc.:0.000ppm

477.0000(-)@18ID#:18 m/z: 477.0000

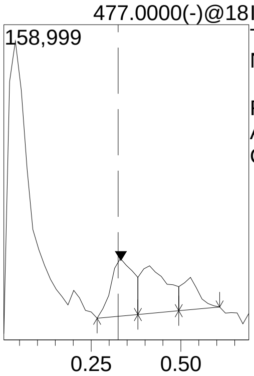

Type:Target
Name:quercetin-3-O- glucuronic acid

Ret.Time:0.333
Area:86236
Conc.:0.000ppm

609.1000(-)@19ID#:19 m/z: 609.1000

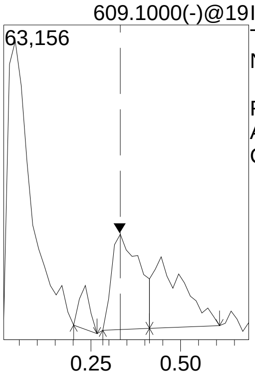

Type:Target
Name:Kaempferol-3-O-glucose

Ret.Time:0.330
Area:63119
Conc.:0.000ppm

609.1000(-)@20ID#:20 m/z: 609.1000

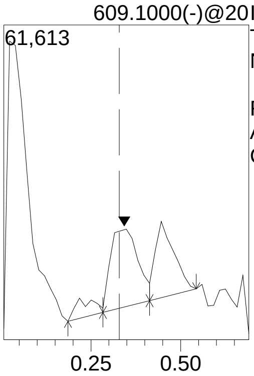

Type:Target
Name:Quercetin-3-O.hexose deoxyhex

Ret.Time:0.343
Area:51593
Conc.:0.000ppm

623.1000(-)@21ID#:21 m/z: 623.1000

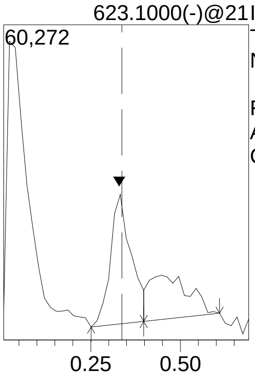

Type:Target
Name:Isorhamnetin- 3-O Rutinoside

Ret.Time:0.329
Area:69035
Conc.:0.000ppm

447.1000(-)@22ID#:22 m/z: 447.1000

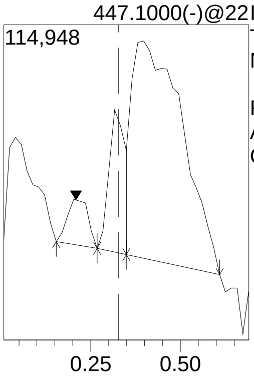

Type:Target
Name:Isorhamnetin-7-O- Pentose

Ret.Time:0.209
Area:32576
Conc.:0.000ppm

447.1000(-)@23

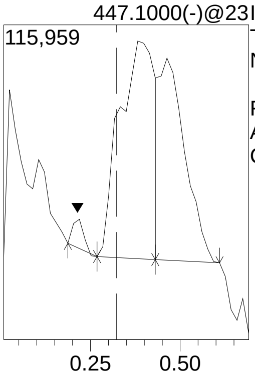

ID#:23 m/z: 447.1000
Type:Target
Name:luteoilin 7-O-glucoside

Ret.Time:0.214
Area:10163
Conc.:0.000ppm

461.1000(-)@24

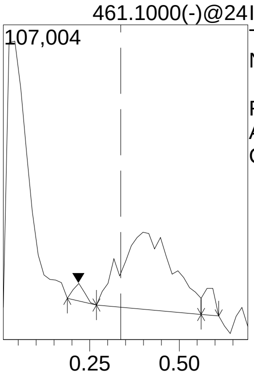

ID#:24 m/z: 461.1000
Type:Target
Name:Kaempferol-3-O-glucuronic acid

Ret.Time:0.218
Area:6655
Conc.:0.000ppm

417.1000(-)@25

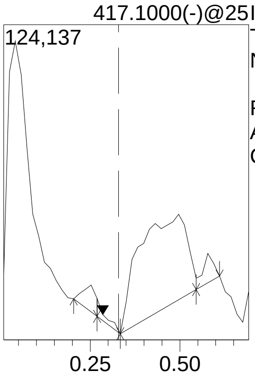

ID#:25 m/z: 417.1000
Type:Target
Name:Kaempferol-3-O-pentose

Ret.Time:0.285
Area:5077
Conc.:0.000ppm

593.1000(-)@26

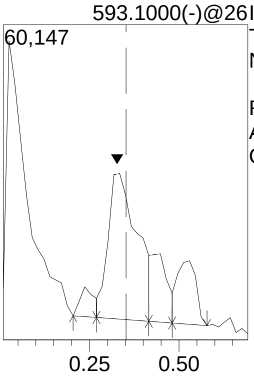

ID#:26 m/z: 593.1000
Type:Target
Name:Kaempferol-3-O-hexose deohyhe

Ret.Time:0.327
Area:94516
Conc.:0.000ppm

153.4000(-)@27

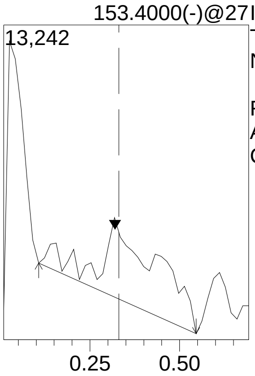

ID#:27 m/z: 153.4000
Type:Target
Name:Tyrosol

Ret.Time:0.320
Area:26411
Conc.:0.000ppm

153.0000(-)@28

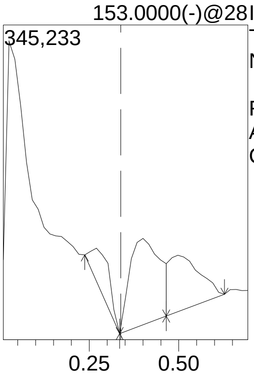

ID#:28 m/z: 153.0000
Type:Target
Name:Protocathecoic acid

Ret.Time:0.000
Area:0
Conc.:N.D.(W/B)

167.0000(-)@29

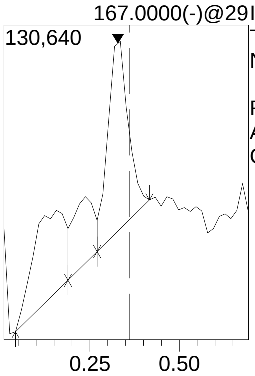

ID#:29 m/z: 167.0000
Type:Target
Name:Vanillic acid

Ret.Time:0.328
Area:178171
Conc.:0.000ppm

197.0000(-)@30

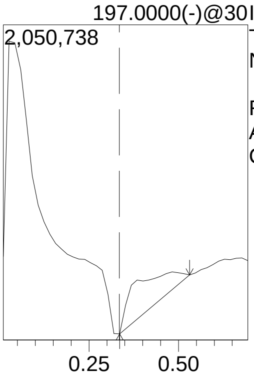

ID#:30 m/z: 197.0000
Type:Target
Name:syringic acid

Ret.Time:0.000
Area:0
Conc.:N.D.(W/B)

137.0000(-)@31ID#:31 m/z: 137.0000

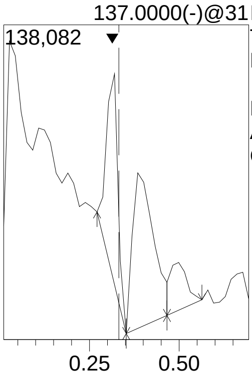

Type:Target
Name:p-hydroxybenzoic\salicilic acid

Ret.Time:0.314
Area:73119
Conc.:0.000ppm

153.0000(-)@32ID#:32 m/z: 153.0000

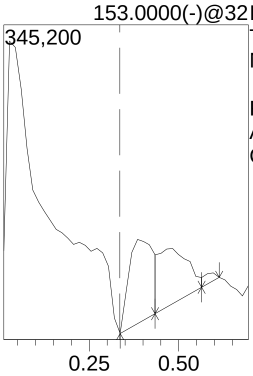

Type:Target
Name:Gentisic acid

Ret.Time:0.000
Area:0
Conc.:N.D.(W/B)

179.0000(-)@33ID#:33 m/z: 179.0000

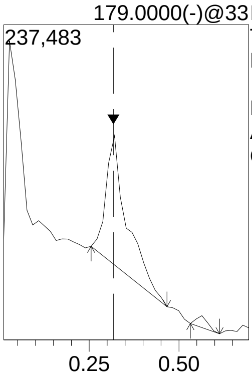

Type:Target
Name:Caffeic acid

Ret.Time:0.317
Area:194990
Conc.:0.000ppm

223.0000(-)@34ID#:34 m/z: 223.0000

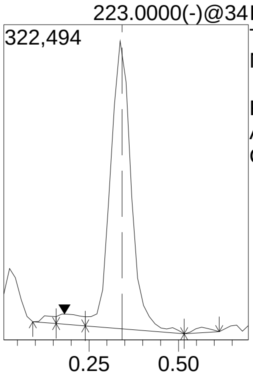

Type:Target
Name:Sinapic acid

Ret.Time:0.181
Area:42203
Conc.:0.000ppm

193.0000(-)@35ID#:35 m/z: 193.0000

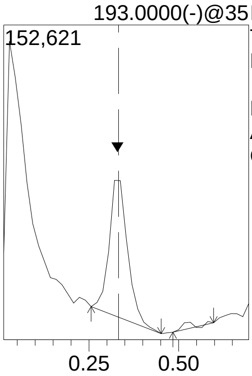

Type:Target
Name:Ferulic acid

Ret.Time:0.329
Area:173798
Conc.:0.000ppm

147.0000(-)@36ID#:36 m/z: 147.0000

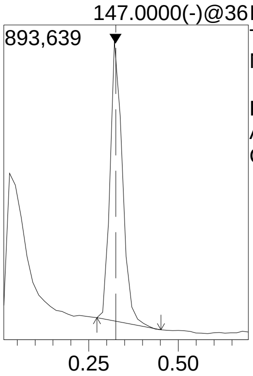

Type:Target
Name:Trans-cinnamic acid

Ret.Time:0.325
Area:1763612
Conc.:0.000ppm

353.0000(-)@37ID#:37 m/z: 353.0000

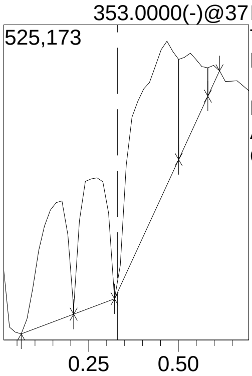

Type:Target
Name:Chlorogenic acid

Ret.Time:0.000
Area:0
Conc.:N.D.(W/B)

289.0000(-)@38ID#:38 m/z: 289.0000

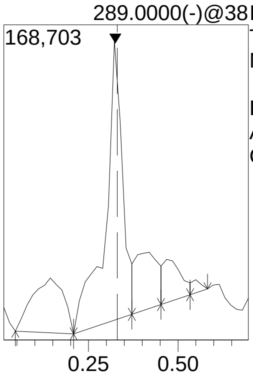

Type:Target
Name:cathechin\epicathechin

Ret.Time:0.325
Area:329139
Conc.:0.000ppm

457.0000(-)@39ID#:39 m/z: 457.0000

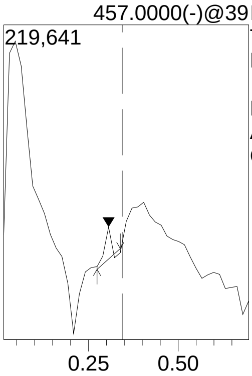

Type:Target
Name:gallocathechin\epigallocathechin

Ret.Time:0.306
Area:13002
Conc.:0.000ppm

305.0000(-)@40ID#:40 m/z: 305.0000

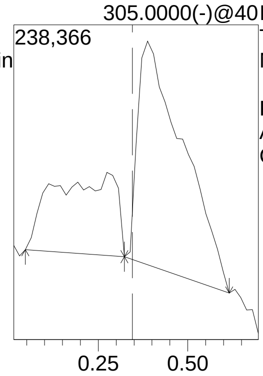

Type:Target
Name:gallocathechin\epigallocathechin

Ret.Time:0.000
Area:0
Conc.:N.D.(W/B)

441.0000(-)@41ID#:41 m/z: 441.0000

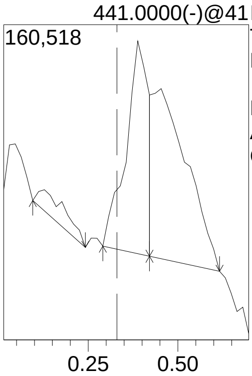

Type:Target
Name:cathechin gallate

Ret.Time:0.000
Area:0
Conc.:N.D.(W/B)

577.0000(-)@42ID#:42 m/z: 577.0000

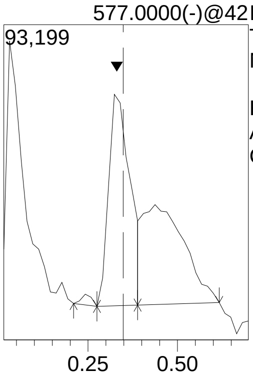

Type:Target
Name:Procianidin

Ret.Time:0.330
Area:142519
Conc.:0.000ppm

317.0000(-)@43ID#:43 m/z: 317.0000

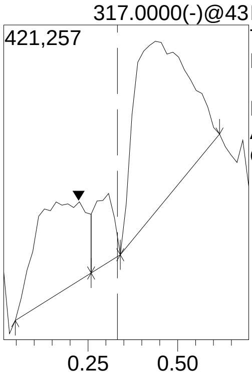

Type:Target
Name:Myricetin

Ret.Time:0.224
Area:516333
Conc.:0.000ppm

285.0000(-)@44ID#:44 m/z: 285.0000

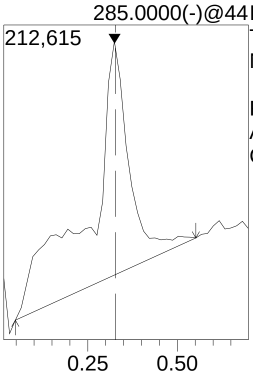

Type:Target
Name:Kaempeferol

Ret.Time:0.325
Area:854532
Conc.:0.000ppm

609.0000(-)@45ID#:45 m/z: 609.0000

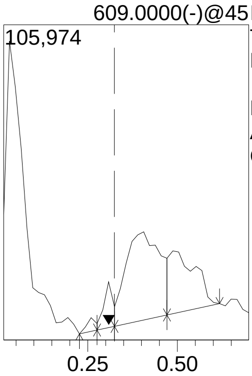

Type:Target
Name:rutin

Ret.Time:0.309
Area:13737
Conc.:0.000ppm

579.0000(-)@46ID#:46 m/z: 579.0000

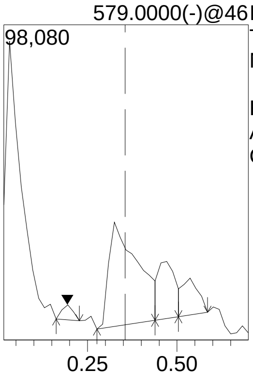

Type:Target
Name:narigin

Ret.Time:0.193
Area:6360
Conc.:0.000ppm
