# Supplementary material for: Phytochemical Analysis, Antispasmodic, Myorelaxant, and Antioxidant Effect of Dysphania ambrosioides (L.) Mosyakin and Clemants Flower Hydroethanolic Extracts and Its Chloroform and Ethyl Acetate Fractions
Source: Molecules. 2021 Dec 1;26(23):7300. doi: 10.3390/molecules26237300 (PMC8659140; doi:10.3390/molecules26237300)
Supplement: Supplementary file 1 [file molecules-26-07300-s001.zip › molecules-1444408-supplementary/File S2_Chloroform extract.pdf]

# = Shimadzu LabSolutions Quant. Browser Data Report =

Acquired by	: System Administrator
Data Acquired	: 23/06/2021 11:09:55
Sample Type	: Unknown
Sample Name	: @4 Extr. CHCl3
Sample ID	:
Sample Amount	: 1
Dilution Factor	: 1
Vial#	: 5
Injection Volume	: 1 uL
Data Filename	: @4 Extr. CHCl3_006.lcd
Method Filename	: polifenoli screening SIM.lcm
Processed by	: System Administrator
Modified Date	: 23/06/2021 11:56:37

198.9000(+)@1ID#:1 m/z: 198.9000

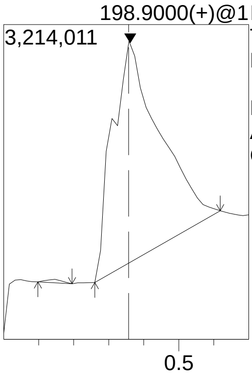

Type:Target
Name:syringic acid SIM

Ret.Time:0.361
Area:21528465
Conc.:0.000ppm

168.9000(-)@2ID#:2 m/z: 168.9000

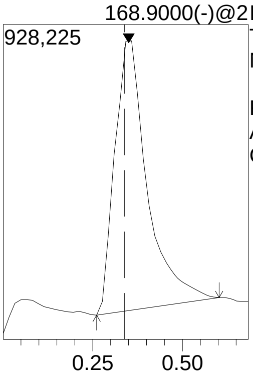

Type:Target
Name:gallic acid SIM

Ret.Time:0.350
Area:4926114
Conc.:0.000ppm

301.0000(-)@3ID#:3 m/z: 301.0000

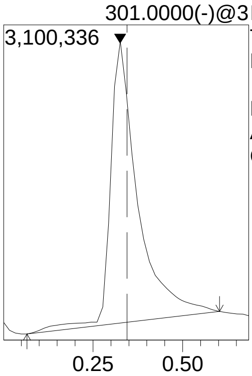

Type:Target
Name:quercetin sim

Ret.Time:0.326
Area:14409964
Conc.:0.000ppm

162.9000(-)@4ID#:4 m/z: 162.9000

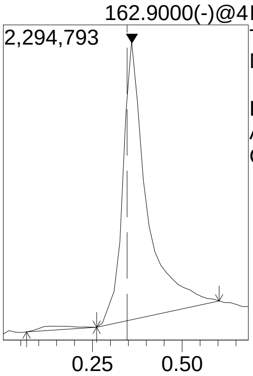

Type:Target
Name:p coumarci acid SIM

Ret.Time:0.360
Area:9310287
Conc.:0.000ppm

303.2000(-)@5ID#:5 m/z: 303.2000

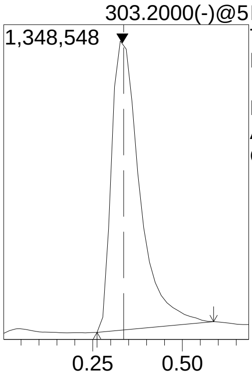

Type:Target
Name:oleochantal SIM

Ret.Time:0.333
Area:6838538
Conc.:0.000ppm

153.0500(-)@6ID#:6 m/z: 153.0500

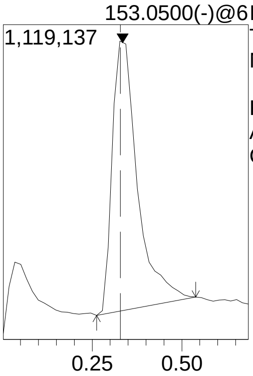

Type:Target
Name:hydroxytyrosol SIM

Ret.Time:0.335
Area:4157569
Conc.:0.000ppm

193.0000(-)@7ID#:7 m/z: 193.0000

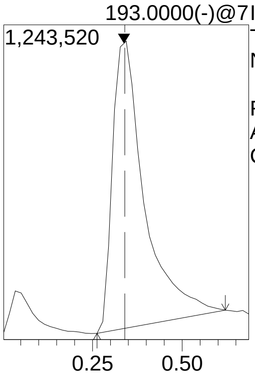

Type:Target
Name:trans ferulic acid SIM

Ret.Time:0.338
Area:6694893
Conc.:0.000ppm

539.0000(-)@8ID#:8 m/z: 539.0000

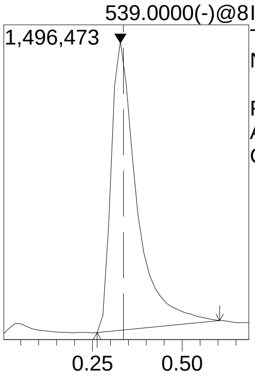

Type:Target
Name:oleuropein SIM

Ret.Time:0.328
Area:6929506
Conc.:0.000ppm

301.3000(-)@9ID#:9 m/z: 301.3000

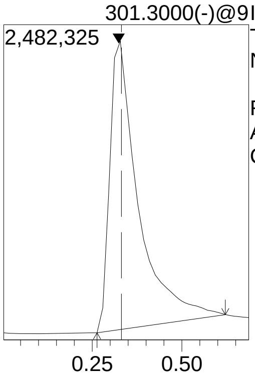

Type:Target
Name:Hesperetin

Ret.Time:0.324
Area:12799161
Conc.:0.000ppm

312.0000(-)@10ID#:10 m/z: 312.0000

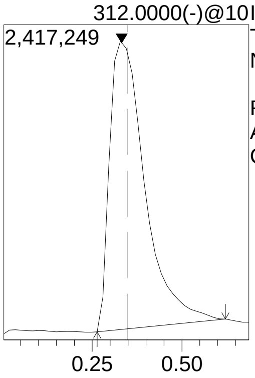

Type:Target
Name:trimethoxyflavone

Ret.Time:0.332
Area:15160348
Conc.:0.000ppm

271.2000(-)@11ID#:11 m/z: 271.2000

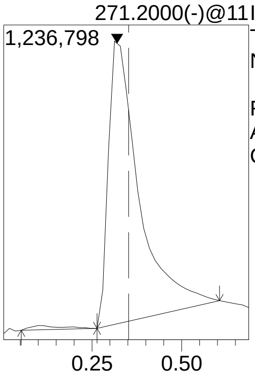

Type:Target
Name:Arbutin

Ret.Time:0.320
Area:6558402
Conc.:0.000ppm

359.0000(-)@12ID#:12 m/z: 359.0000

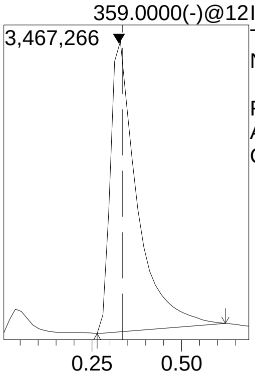

Type:Target
Name:Rosmarinic acid

Ret.Time:0.325
Area:16761411
Conc.:0.000ppm

455.0000(-)@13ID#:13 m/z: 455.0000

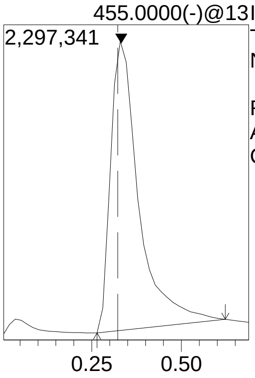

Type:Target
Name:ursolic acid

Ret.Time:0.332
Area:11568299
Conc.:0.000ppm

269.0000(-)@14ID#:14 m/z: 269.0000

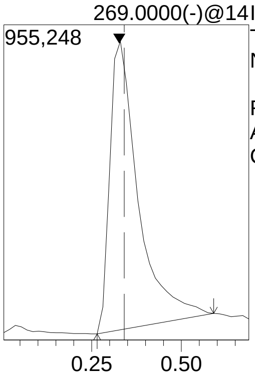

Type:Target
Name:Apigenin

Ret.Time:0.327
Area:4720190
Conc.:0.000ppm

537.1000(-)@15ID#:15 m/z: 537.1000

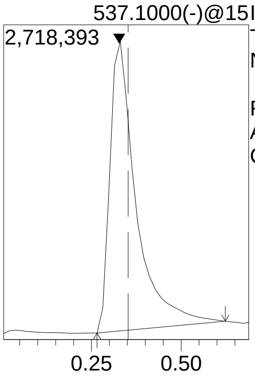

Type:Target
Name:Amentoflavone

Ret.Time:0.327
Area:12890487
Conc.:0.000ppm

284.9000(-)@16ID#:16 m/z: 284.9000

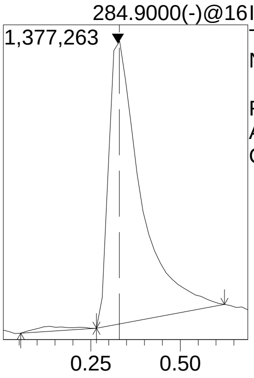

Type:Target
Name:luteoilin

Ret.Time:0.326
Area:7645208
Conc.:0.000ppm

463.1000(-)@17ID#:17 m/z: 463.1000

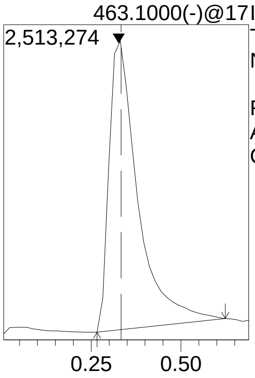

Type:Target
Name:quercetin-3-O-glucoside

Ret.Time:0.326
Area:12986165
Conc.:0.000ppm

477.0000(-)@18ID#:18 m/z: 477.0000

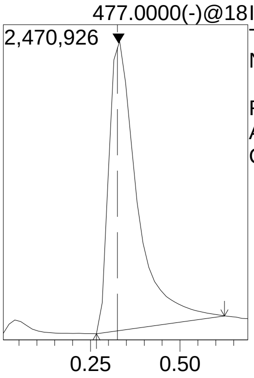

Type:Target
Name:quercetin-3-O- glucuronic acid

Ret.Time:0.328
Area:12686988
Conc.:0.000ppm

609.1000(-)@19ID#:19 m/z: 609.1000

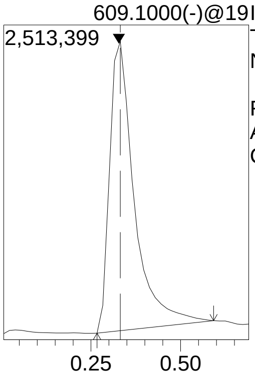

Type:Target
Name:Kaempferol-3-O-glucose

Ret.Time:0.328
Area:11219929
Conc.:0.000ppm

609.1000(-)@20ID#:20 m/z: 609.1000

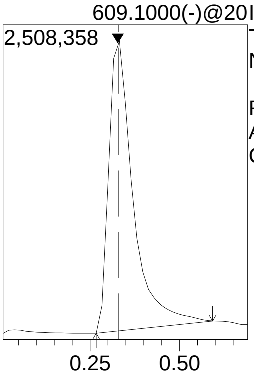

Type:Target
Name:Quercetin-3-O.hexose deoxyhex

Ret.Time:0.328
Area:11154310
Conc.:0.000ppm

623.1000(-)@21ID#:21 m/z: 623.1000

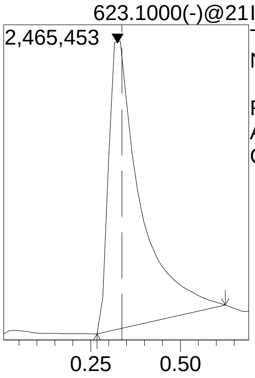

Type:Target
Name:Isorhamnetin- 3-O Rutinoside

Ret.Time:0.325
Area:14197119
Conc.:0.000ppm

447.1000(-)@22ID#:22 m/z: 447.1000

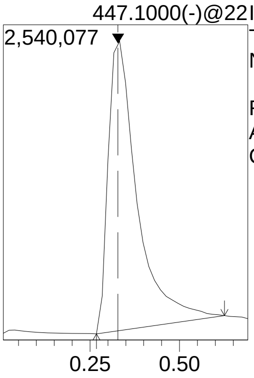

Type:Target
Name:Isorhamnetin-7-O- Pentose

Ret.Time:0.328
Area:13247418
Conc.:0.000ppm

447.1000(-)@23ID#:23 m/z: 447.1000

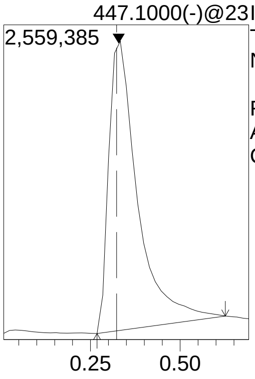

Type:Target
Name:luteoilin 7-O-glucoside

Ret.Time:0.328
Area:13269900
Conc.:0.000ppm

461.1000(-)@24ID#:24 m/z: 461.1000

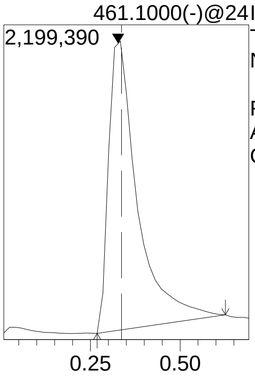

Type:Target
Name:Kaempferol-3-O-glucuronic acid

Ret.Time:0.327
Area:11403805
Conc.:0.000ppm

417.1000(-)@25ID#:25 m/z: 417.1000

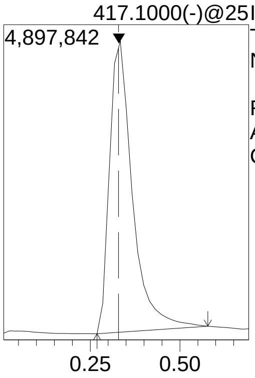

Type:Target
Name:Kaempferol-3-O-pentose

Ret.Time:0.330
Area:20374346
Conc.:0.000ppm

593.1000(-)@26ID#:26 m/z: 593.1000

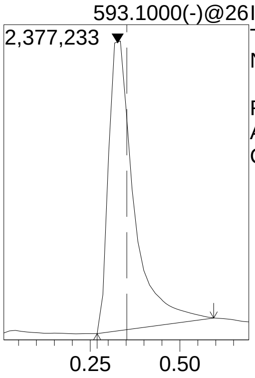

Type:Target
Name:Kaempferol-3-O-hexose deohyh[illegible]

Ret.Time:0.326
Area:11000606
Conc.:0.000ppm

153.4000(-)@27ID#:27 m/z: 153.4000

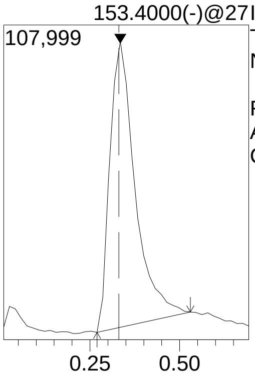

Type:Target
Name:Tyrosol

Ret.Time:0.334
Area:464518
Conc.:0.000ppm

153.0000(-)@28ID#:28 m/z: 153.0000

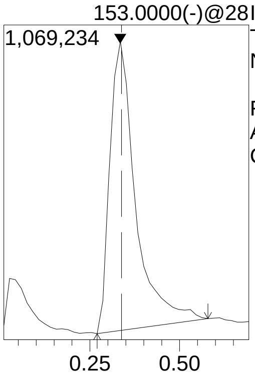

Type:Target
Name:Protocathecoic acid

Ret.Time:0.334
Area:4132216
Conc.:0.000ppm

167.0000(-)@29ID#:29 m/z: 167.0000

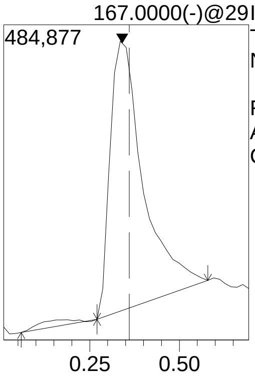

Type:Target
Name:Vanillic acid

Ret.Time:0.341
Area:2502852
Conc.:0.000ppm

197.0000(-)@30ID#:30 m/z: 197.0000

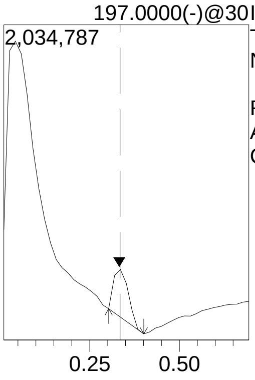

Type:Target
Name:syringic acid

Ret.Time:0.332
Area:689323
Conc.:0.000ppm

137.0000(-)@31 ID#:31 m/z: 137.0000

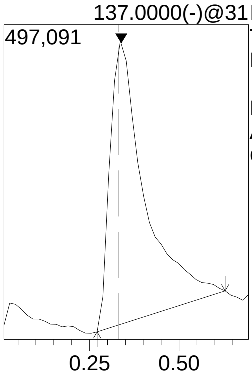

Type:Target
Name:p-hydroxybenzoic\salicilic acid

Ret.Time:0.339
Area:2544256
Conc.:0.000ppm

153.0000(-)@32 ID#:32 m/z: 153.0000

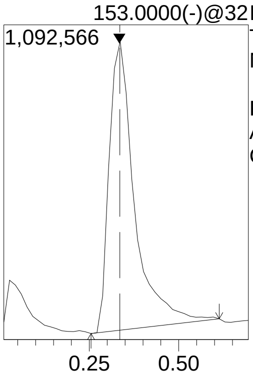

Type:Target
Name:Gentisic acid

Ret.Time:0.334
Area:4170821
Conc.:0.000ppm

179.0000(-)@33 ID#:33 m/z: 179.0000

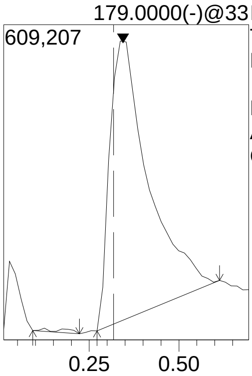

Type:Target
Name:Caffeic acid

Ret.Time:0.344
Area:2905842
Conc.:0.000ppm

223.0000(-)@34 ID#:34 m/z: 223.0000

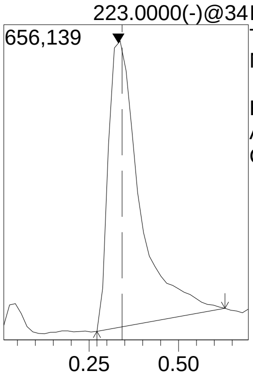

Type:Target
Name:Sinapic acid

Ret.Time:0.332
Area:3474189
Conc.:0.000ppm

193.0000(-)@35 ID#:35 m/z: 193.0000

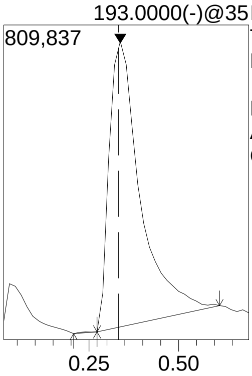

Type:Target
Name:Ferulic acid

Ret.Time:0.337
Area:4233464
Conc.:0.000ppm

147.0000(-)@36 ID#:36 m/z: 147.0000

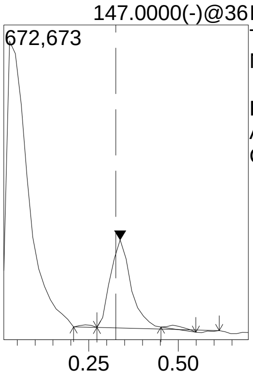

Type:Target
Name:Trans-cinnamic acid

Ret.Time:0.338
Area:639045
Conc.:0.000ppm

353.0000(-)@37 ID#:37 m/z: 353.0000

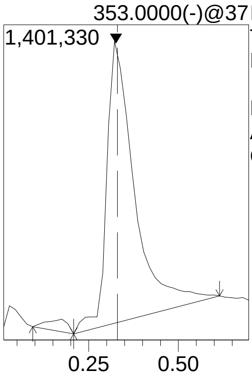

Type:Target
Name:Chlorogenic acid

Ret.Time:0.326
Area:6396078
Conc.:0.000ppm

289.0000(-)@38 ID#:38 m/z: 289.0000

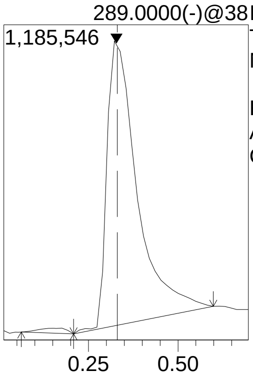

Type:Target
Name:cathechin\epicathechin

Ret.Time:0.328
Area:6207884
Conc.:0.000ppm

457.0000(-)@39ID#:39 m/z: 457.0000

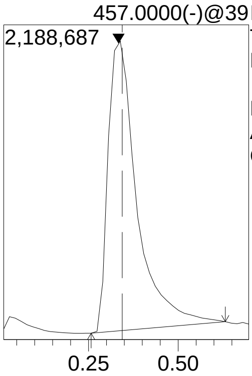

Type:Target
Name:gallocathechin\epigallocathechin

Ret.Time:0.334
Area:11164330
Conc.:0.000ppm

305.0000(-)@40ID#:40 m/z: 305.0000

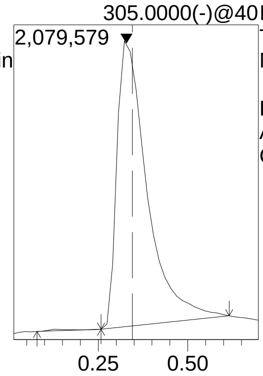

Type:Target
Name:gallocathechin\epigallocathechin

Ret.Time:0.329
Area:10607470
Conc.:0.000ppm

441.0000(-)@41ID#:41 m/z: 441.0000

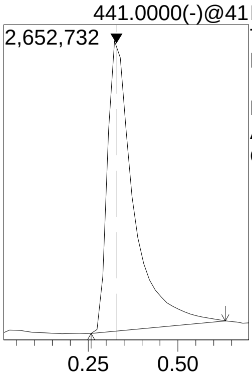

Type:Target
Name:cathechin gallate

Ret.Time:0.329
Area:12511204
Conc.:0.000ppm

577.0000(-)@42ID#:42 m/z: 577.0000

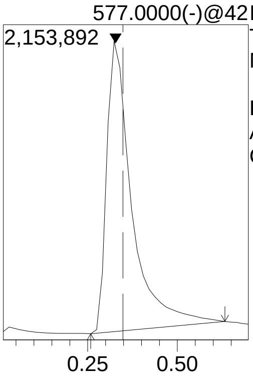

Type:Target
Name:Procianidin

Ret.Time:0.328
Area:9792735
Conc.:0.000ppm

317.0000(-)@43ID#:43 m/z: 317.0000

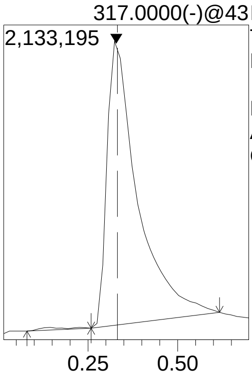

Type:Target
Name:Myricetin

Ret.Time:0.329
Area:10874754
Conc.:0.000ppm

285.0000(-)@44ID#:44 m/z: 285.0000

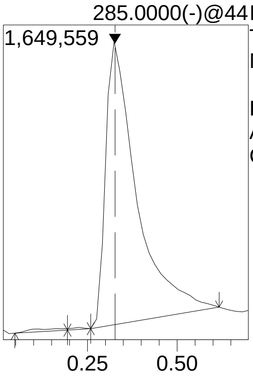

Type:Target
Name:Kaempeferol

Ret.Time:0.326
Area:8846690
Conc.:0.000ppm

609.0000(-)@45ID#:45 m/z: 609.0000

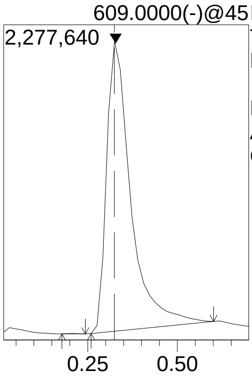

Type:Target
Name:rutin

Ret.Time:0.328
Area:9919015
Conc.:0.000ppm

579.0000(-)@46ID#:46 m/z: 579.0000

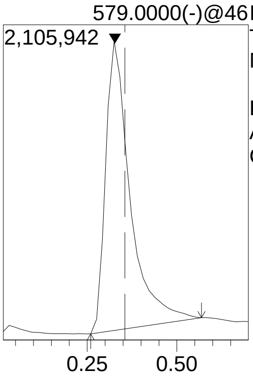

Type:Target
Name:narigin

Ret.Time:0.327
Area:9302456
Conc.:0.000ppm
